# Significant National Declines in Neurosurgical Intervention for Mild Traumatic Brain Injury with Intracranial Hemorrhage: A 13-Year Review of the National Trauma Data Bank

**DOI:** 10.1089/neur.2022.0077

**Published:** 2023-03-17

**Authors:** Alessandro Orlando, Josef Coresh, Matthew M. Carrick, Glenda Quan, Gina M. Berg, Laxmi Dhakal, David Hamilton, Robert Madayag, Carlos H. Palacio Lascano, David Bar-Or

**Affiliations:** ^1^ Johns Hopkins Bloomberg School of Public Health, Johns Hopkins University, Baltimore, Maryland, USA.; ^2^ Medical City Plano, Plano, Texas, USA.; ^3^ Swedish Medical Center, Englewood, Colorado, USA.; ^4^ Wesley Medical Center, Wichita, Kansas, USA.; ^5^ Penrose Hospital, Colorado Springs, Colorado, USA.; ^6^ St. Anthony Hospital, Lakewood, Colorado, USA.; ^7^ South Texas Health System McAllen, McAllen, Texas, USA.

**Keywords:** epidemiology, intracranial hemorrhage, mild, National Trauma Data Bank, neurosurgical intervention, time, traumatic brain injury

## Abstract

There have been large changes over the past several decades to patient demographics in those presenting with mild traumatic brain injury (mTBI) with intracranial hemorrhage (ICH; complicated mTBI) with the potential to affect the use of neurosurgical interventions. The objective of this study was to characterize long-term trends of neurosurgical interventions in patients with complicated mTBI using 13 years of the National Trauma Data Bank (NTDB). This was a retrospective cohort study of adult (≥18 years) trauma patients included in the NTDB from 2007 to 2019 who had an emergency department Glasgow Coma Scale score 13–15, an intracranial hemorrhage (ICH), and no skull fracture. Neurosurgical intervention time trends were quantified for each ICH type using mixed-effects logistic regression with random slopes and intercepts for hospitals, as well as covariates for time and 14 demographic, injury, and hospital characteristics. In total, 666,842 ICH patients across 1060 hospitals were included. The four most common hemorrhages were isolated subdural hemorrhage (36%), isolated subarachnoid hemorrhage (24%), multiple hemorrhage types (24%), and isolated unspecified hemorrhages (9%). Overall, 49,220 (7%) patients received a neurosurgical intervention. After adjustment, the odds of neurosurgical intervention significantly decreased every 10 years by the following odds ratios (odds ratio [95% confidence interval]): 0.85 [0.78, 0.93] for isolated subdural, 0.63 [0.51, 0.77] for isolated subarachnoid, 0.50 [0.41, 0.62] for isolated unspecified, and 0.79 [0.73, 0.86] for multiple hemorrhages. There were no significant temporal trends in neurosurgical intervention odds for isolated epidural hemorrhages (0.87 [0.68, 1.12]) or isolated contusions/lacerations (1.03 [0.75, 1.41]). In the setting of complicated mTBI, the four most common ICH types were associated with significant declines in the odds of neurosurgical intervention over the past decade. It remains unclear whether changing hemorrhage characteristics or practice patterns drove these trends.

## Introduction

Mild traumatic brain injuries (mTBIs) are a highly prevalent and important public health injury. This injury is defined as a traumatic brain injury with a Glasgow Coma Scale (GCS) 13–15 and <30 min of loss of consciousness. Nearly 2.4 million mTBIs are seen in an emergency department or hospital or cause death in the United States each year.^1–3^ Though considered “mild,” nearly 10% of these brain injuries are accompanied by potentially fatal intracranial hemorrhages (ICHs), also known as complicated mTBIs.^2,4^ Once an ICH is identified on initial head imaging, a neurosurgical consultation is obtained because of the potential need for neurosurgical intervention. If the initial facility lacks neurosurgical coverage, then the patient is transferred to the nearest facility with neurosurgical coverage. This workup of patients with complicated mTBI occurs at ∼16,000 per month in the United States. Complicated mTBI is a serious problem that is likely to get worse in the coming decades.

The United States has seen a trend toward an increasing number of TBIs in recent years.^5–8^ One reason to expect that the number of TBIs will increase is because advanced age is a strong risk factor for mTBI^2,9^ and because the population with advanced age is rapidly increasing.^10^ The U.S. Census Bureau estimates that, in 2060, persons ≥65 years of age will comprise nearly 25% of the population (94.7 million), equating to a 92% increase in population size compared to 2016 (49.2 million).^11^ This doubling in the population most at risk for mTBI highlights the need to begin to consider how changing population demographics has affected healthcare delivery up to the present, and how it might be affected in the future.

From the perspective of complicated mTBI, an important outcome to consider being affected by time, changing patient demographics, and limited neurosurgical personnel is neurosurgical interventions. Each neurosurgeon decides whether to pursue surgical management of an ICH, with their decisions guided by a combination of surgical experience and surgical guidelines. However, surgical management guidelines for mTBI are limited by non-descript language or supported with older studies with small sample sizes.^12,13^ Therefore, in practice, more decisional weight is placed on clinical and surgical experience than on surgical guidelines. Reliance on personal experience might allow for gradual changes to neurosurgical decision making over time, particularly when combined with a large change in patient demographics.

The objectives of this study were to investigate ICH-specific national long-term trends of neurosurgical interventions in patients with complicated mTBI in the United States, and how these trends were impacted by changing demographic, injury, and hospital characteristics.

## Methods

This was a retrospective cohort study using deidentified, publicly available data from the American College of Surgeons (ACS) National Trauma Data Bank (NTDB). All patients from 2007 to 2019 were included who met the following criteria: age 18–89 (ages <18 and >89 were redacted in NTDB because of privacy reasons and thus excluded from the current study); emergency department GCS score 13–15; presented with at least one of the following traumatic ICHs: International Classification of Diseases, Ninth Revision (ICD-9) and Tenth Revision (ICD-10) diagnosis codes: contusion/laceration of parenchyma or cerebellum, subdural hemorrhage (SDH), epidural hemorrhage (EDH), subarachnoid hemorrhage (SAH), or unspecified ICH; diagnosis codes only examined initial encounter codes; no skull fracture; and no missing covariate data (see below). Because these data were publicly available and do not contain identifiable information, this study was not considered human subjects research by the National Institutes of Health^14^ and Department of Health and Human Services.^15^

### Covariates and outcome

The following demographic covariates were used: year of hospital admission; age (years); biological sex (male, female); self-identified race (Asian, Black, White, or Other); and payment method (Medicaid, Medicare, private/commercial, or self-pay). The three hospital covariates were: anonymized hospital identifier; hospital bed size (≤200, 201–400, 401–600, and >600); and ACS trauma designation level (1, 2, 3, 4, or undesignated). Finally, the injury covariates were: number and type of ICH (ICD-9 and -10 diagnosis codes for isolated contusion/laceration of parenchyma or cerebellum, isolated SDH, isolated EDH, isolated SAH, isolated unspecified ICH, and multiple types of ICHs); interhospital transfer (yes/no); mechanism of injury (fall, motorcycle accident, motor vehicle accident, struck by/against, transport [other], and other); emergency department GCS score; Injury Severity Score [ISS]; injury type (blunt, penetrating, burn, and other/unspecified); intent of injury (assault, self-inflicted, unintentional, undetermined, and other/unspecified); coagulopathy (presence of pre-injury anticoagulant, antiplatelet [excluding chronic aspirin use], thrombin inhibitor, or thrombolytic agent); and emergency department vital signs: systolic blood pressure (mm Hg), pulse (beats/min), and respiratory rate (breaths/min).

The primary outcome for this study was a documented neurosurgical intervention (e.g., craniotomy, craniectomy, intracranial pressure [ICP] monitor placement, and ventriculostomy), determined by ICD-9 and -10 procedure codes. All ICD-10 procedure codes were converted to ICD-9 procedure codes using the yearly general equivalence mappings provided by the Center for Medicare and Medicaid Services.^16^ We defined neurosurgical procedures using the ICD-9 codes 1.00–2.99, which include craniotomies, craniectomies, burr holes, ICP monitors, ventriculostomies, and ventricular shunts. See the Supplementary Material for a full mapping of all 1448 ICD-10 neurosurgical intervention procedure codes used to map to the ICD-9 coding scheme. The secondary outcome was a composite of in-hospital mortality or hospital discharge to hospice.

### Statistical analyses

Descriptive characteristics were summarized using frequencies and percentages, means and standard deviations (SDs), and medians and interquartile ranges. No formal hypothesis testing was done on descriptive statistics over time or between neurosurgical intervention groups. A descriptive graph was created showing the average number of study subjects admitted to each hospital over time, stratified by ICH type. These graph data were adjusted for average subject age in a linear regression model with a dependent variable of average patients per hospital and independent variables of year of admission and average subject age; slopes were adjusted for an age of 55 years.

The main analysis of change in neurosurgical interventions over time used a mixed-effects logistic regression model with random intercepts and slopes for each hospital using an unstructured covariance model; the fixed effects were all other covariates previously outlined, except for ICH type. Non-linear associations of each variable with time were evaluated using locally estimated scatterplot smoothing plots. Each ICH type was modeled separately using an iterative, five-model approach. Model 1 tested the overall trend for neurosurgical interventions (over all ICH types) across the study time frame. Next, model 1 was stratified by hemorrhage type (model 2). Model 3 added demographic covariates, model 4 added hospital covariates, and model 5 added injury covariates. This modeling approach allowed for an understanding of how the probability of neurosurgical intervention changed over time for individual ICH types and how each was affected by potential confounding categories (i.e., demographic, hospital, and injury characteristics), and incorporated longitudinal data (repeated measures) from each hospital. A similar modeling technique was used to examine trends in in-hospital mortality or discharge to hospice. All analyses used SAS (version 9.4; SAS Institute Inc., Cary, NC), with two-tailed hypothesis tests with an alpha of 0.05.

## Results

There were 10,860,952 patients in the NTDB across the 13-year study period. Overall, 2,015,988 patients were excluded because of age <18 years or redacted age; 1,288,799 patients were excluded for an emergency department GCS score <13; 6,577,548 patients were excluded for not having an ICH; 220,351 patients were excluded for presenting with a skull fracture; and 91,424 patients were excluded for missing covariate values. The final analysis population consisted of 666,842 patients across 1060 hospitals. The mean (SD) number of years contributed by each hospital was 7.35 (4.19) years, and the mean (SD) number of patients contributed by each hospital was 629.10 (849.49). Regarding ICHs, the most frequent hemorrhage type was isolated SDHs (35.75%; *n* = 238,419), followed by multiple hemorrhages (23.88%; *n* = 159,220), isolated SAHs (23.61%; *n* = 157,414), isolated unspecified hemorrhages (9.04%; *n* = 60,315), isolated lacerations and contusions (6.55%; *n* = 43,695), and isolated EDHs (1.17%; *n* = 7779). In patients with multiple hemorrhages, 79% had an SAH, 74% had an SDH, 35% had an unspecified hemorrhage, 30% had laceration or contusion, and 4% had an EDH.

**FIG. 1.**
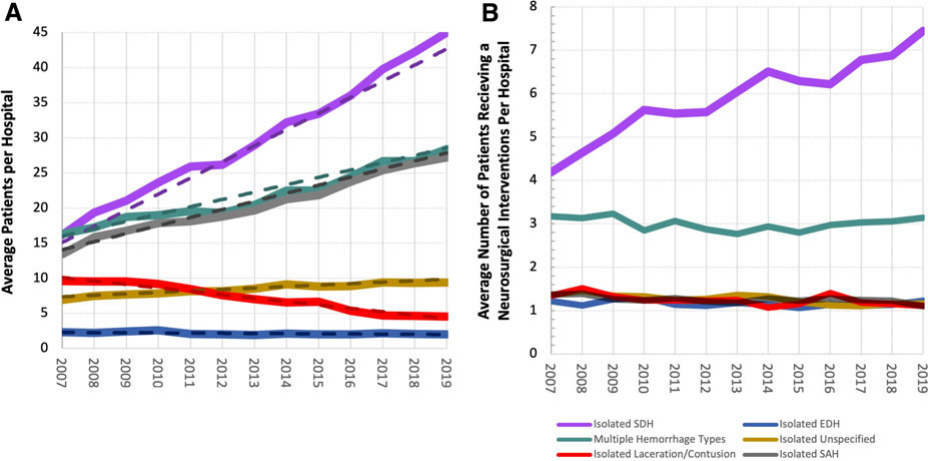
Changes to yearly admissions and neurosurgical interventions in patients with complicated mTBI, stratified by ICH. (**A**) Long-term trends in average number of complicated mTBI patients admitted to each hospital by ICH type. Solid lines indicate observed data, and dashed lines indicate trends adjusted for average patient age. Isolated subdural hemorrhages had the largest absolute increase in average number of patients admitted per hospital, whereas isolated lacerations/contusions had the steepest decrease in average patients per hospital. Adjusting for average patient age minimally explained the trends observed in the observed data. (**B**) Long-term trends in average number of patients receiving a neurosurgical intervention per hospital, stratified by ICH type. Isolated SDHs were the only hemorrhage type to show increasing trends over time; the remaining hemorrhage types show stable trends over time. EDH, epidural hemorrhage; ICH, intracranial hemorrhage; SAH, subarachnoid hemorrhage; SDH, subdural hemorrhage.

Overall, the number of patients included in each year increased as a result of more hospitals contributing data and more patients per hospital (Fig. 1A, Supplementary Fig. S1); Figure 1A also shows that average patient age explained very little of the change in average number of patients per hospital over time. Table 1 describes how the study population characteristics changed over the 13-year study period. There were increases in the mean age (57–66 years), proportion paying with Medicare (36–54%), fall-related injuries (48–70%), unintentional injuries (89–94%), and comorbid coagulopathy (5–23%). Meanwhile, there were decreases in proportion of male (62–56%), the proportion of patients using self-pay (16–6%), motor-vehicle-occupant–related injuries (23–12%), mean ISS (18.04–14.01), and in the proportion of undesignated trauma centers contributing data (40–25%).

**Table 1.** Demographic, Hospital, and Injury Characteristics of Study Population Over 13-Year Study Period

Variable,* n *(%)	Overall	2007	2008	2009	2010	2011	2012	2013	2014	2015	2016	2017	2018	2019
	666,842 (100%)	16,433 (2%)	27,381 (4%)	34,201 (5%)	38,626 (6%)	45,034 (7%)	48,460 (7%)	50,562 (8%)	54,330 (8%)	60,112 (9%)	65,646 (10%)	71,139 (11%)	75,904 (11%)	79,014 (12%)
Age, years, mean (SD)	63.3 (19.44)	57.07 (21.23)	58.47 (21.06)	59.73 (20.70)	60.78 (20.48)	61.83 (20.07)	62.27 (19.97)	63.29 (19.54)	63.8 (19.20)	63.83 (19.26)	63.77 (19.07)	64.79 (18.67)	65.54 (18.23)	65.91 (17.96)
Male	383,749 (58%)	10,160 (62%)	16,638 (61%)	20,203 (59%)	22,738 (59%)	26,095 (58%)	28,012 (58%)	28,814 (57%)	31,085 (57%)	34,263 (57%)	37,704 (57%)	40,600 (57%)	42,889 (57%)	44,548 (56%)
Race														
Asian	16,575 (2%)	310 (2%)	609 (2%)	748 (2%)	823 (2%)	1017 (2%)	1112 (2%)	1105 (2%)	1290 (2%)	1515 (3%)	1790 (3%)	1958 (3%)	2078 (3%)	2220 (3%)
Black	60,233 (9%)	1722 (10%)	2644 (10%)	3187 (9%)	3500 (9%)	4053 (9%)	4275 (9%)	4350 (9%)	4678 (9%)	5399 (9%)	5889 (9%)	6468 (9%)	6762 (9%)	7306 (9%)
Other	50,897 (8%)	1503 (9%)	2596 (9%)	2632 (8%)	3019 (8%)	3414 (8%)	3707 (8%)	3626 (7%)	3834 (7%)	4065 (7%)	5323 (8%)	5736 (8%)	5521 (7%)	5921 (7%)
White	539,137 (81%)	12,898 (78%)	21,532 (79%)	27,634 (81%)	31,284 (81%)	36,550 (81%)	39,366 (81%)	41,481 (82%)	44,528 (82%)	49,133 (82%)	52,644 (80%)	56,977 (80%)	61,543 (81%)	63,567 (80%)
Payment method														
Medicaid	55,388 (8%)	1173 (7%)	1948 (7%)	2471 (7%)	2814 (7%)	3116 (7%)	3296 (7%)	3387 (7%)	4525 (8%)	5382 (9%)	6512 (10%)	6906 (10%)	6906 (9%)	6952 (9%)
Medicare	323,920 (49%)	5956 (36%)	10,416 (38%)	13,941 (41%)	16,664 (43%)	20,288 (45%)	22,560 (47%)	24,568 (49%)	26,999 (50%)	30,231 (50%)	32,809 (50%)	36,565 (51%)	40,437 (53%)	42,486 (54%)
Other	58,654 (9%)	2543 (15%)	4230 (15%)	4961 (15%)	5131 (13%)	6000 (13%)	6326 (13%)	6667 (13%)	6616 (12%)	3018 (5%)	3169 (5%)	3314 (5%)	3388 (4%)	3291 (4%)
Private/commercial	170,868 (26%)	4104 (25%)	7069 (26%)	8499 (25%)	9322 (24%)	10,515 (23%)	11,266 (23%)	11,047 (22%)	11,879 (22%)	17,089 (28%)	18,633 (28%)	19,769 (28%)	20,344 (27%)	21,332 (27%)
Self	58,012 (9%)	2657 (16%)	3718 (14%)	4329 (13%)	4695 (12%)	5115 (11%)	5012 (10%)	4893 (10%)	4311 (8%)	4392 (7%)	4523 (7%)	4585 (6%)	4829 (6%)	4953 (6%)
Interhospital transfer	257,314 (39%)	5719 (35%)	9410 (34%)	12,693 (37%)	14,930 (39%)	17,258 (38%)	18,421 (38%)	20,057 (40%)	21,501 (40%)	23,402 (39%)	24,954 (38%)	27,939 (39%)	29,895 (39%)	31,135 (39%)
Hospital beds														
≤200	42,676 (6%)	715 (4%)	1436 (5%)	1769 (5%)	2363 (6%)	2795 (6%)	3072 (6%)	3457 (7%)	3389 (6%)	3625 (6%)	3788 (6%)	5332 (7%)	5427 (7%)	5508 (7%)
201–400	185,132 (28%)	4240 (26%)	6801 (25%)	8512 (25%)	9765 (25%)	11,043 (25%)	12,560 (26%)	13,806 (27%)	14,194 (26%)	16,348 (27%)	18,289 (28%)	21,695 (30%)	23,369 (31%)	24,510 (31%)
401–600	186,245 (28%)	5179 (32%)	8008 (29%)	10,036 (29%)	11,468 (30%)	11,980 (27%)	13,539 (28%)	13,831 (27%)	16,012 (29%)	16,552 (28%)	18,090 (28%)	18,826 (26%)	20,852 (27%)	21,872 (28%)
>600	252,789 (38%)	6299 (38%)	11,136 (41%)	13,884 (41%)	15,030 (39%)	19,216 (43%)	19,289 (40%)	19,468 (39%)	20,735 (38%)	23,587 (39%)	25,479 (39%)	25,286 (36%)	26,256 (35%)	27,124 (34%)
ACS trauma level designation														
1	251,129 (38%)	5880 (36%)	10,418 (38%)	12,700 (37%)	14,113 (37%)	15,951 (35%)	16,886 (35%)	16,522 (33%)	18,092 (33%)	20,150 (34%)	23,143 (35%)	31,164 (44%)	32,502 (43%)	33,608 (43%)
2	154,662 (23%)	3553 (22%)	6189 (23%)	7156 (21%)	8285 (21%)	10,035 (22%)	10,267 (21%)	10,395 (21%)	11,218 (21%)	13,172 (22%)	15,143 (23%)	17,634 (25%)	20,059 (26%)	21,556 (27%)
3 or 4	23,492 (4%)	433 (3%)	635 (2%)	1069 (3%)	1141 (3%)	1302 (3%)	1388 (3%)	1176 (2%)	1473 (3%)	1809 (3%)	1787 (3%)	3413 (5%)	3766 (5%)	4100 (5%)
NA/undesignated	237,559 (36%)	6567 (40%)	10,139 (37%)	13,276 (39%)	15,087 (39%)	17,746 (39%)	19,919 (41%)	22,469 (44%)	23,547 (43%)	24,981 (42%)	25,573 (39%)	18,928 (27%)	19,577 (26%)	19,750 (25%)
Mechanism of injury														
Fall	478,313 (63%)	12,432 (48%)	18,945 (52%)	24,270 (56%)	27,575 (58%)	31,524 (60%)	34,111 (61%)	36,982 (64%)	39,765 (64%)	43,623 (65%)	45,088 (64%)	50,403 (67%)	55,205 (69%)	58,390 (70%)
MVT motorcyclist	25,769 (3%)	1280 (5%)	1819 (5%)	1772 (4%)	1870 (4%)	2043 (4%)	2222 (4%)	1988 (3%)	2181 (4%)	2309 (3%)	2100 (3%)	2037 (3%)	1987 (2%)	2161 (3%)
MVT occupant	110,891 (15%)	5795 (23%)	7229 (20%)	7923 (18%)	7893 (17%)	8105 (15%)	8517 (15%)	8393 (14%)	8496 (14%)	9122 (14%)	9340 (13%)	9873 (13%)	10,051 (13%)	10,154 (12%)
Other	72,633 (10%)	2917 (11%)	3839 (11%)	4236 (10%)	4612 (10%)	5013 (10%)	5199 (9%)	5276 (9%)	5886 (9%)	6132 (9%)	7930 (11%)	7398 (10%)	7158 (9%)	7037 (8%)
Struck by, against	51,071 (7%)	2188 (9%)	2960 (8%)	3468 (8%)	3554 (8%)	3779 (7%)	4040 (7%)	3801 (7%)	3865 (6%)	4103 (6%)	4840 (7%)	4837 (6%)	4729 (6%)	4907 (6%)
Transport, other	19,589 (3%)	1084 (4%)	1497 (4%)	1666 (4%)	1737 (4%)	1846 (4%)	1905 (3%)	1774 (3%)	1778 (3%)	1905 (3%)	1173 (2%)	1079 (1%)	1048 (1%)	1097 (1%)
No. of intracranial hemorrhages														
1	577,050 (76%)	19,234 (75%)	27,689 (76%)	32,928 (76%)	36,222 (77%)	40,073 (77%)	43,052 (77%)	44,852 (77%)	47,257 (76%)	51,347 (76%)	53,264 (76%)	56,996 (75%)	60,844 (76%)	63,292 (76%)
2	143,710 (19%)	5004 (19%)	6706 (18%)	8064 (19%)	8707 (18%)	9653 (18%)	10,252 (18%)	10,608 (18%)	11,639 (19%)	12,558 (19%)	13,755 (20%)	14,892 (20%)	15,527 (19%)	16,345 (20%)
3	33,401 (4%)	1278 (5%)	1647 (5%)	2055 (5%)	2000 (4%)	2270 (4%)	2384 (4%)	2447 (4%)	2755 (4%)	2960 (4%)	3104 (4%)	3402 (4%)	3423 (4%)	3676 (4%)
4	3924 (1%)	169 (1%)	232 (1%)	278 (1%)	300 (1%)	299 (1%)	295 (1%)	300 (1%)	309 (0.5%)	312 (0.5%)	330 (0.5%)	322 (0.4%)	362 (0.5%)	416 (0.5%)
5	180 (0%)	11 (0%)	15 (0%)	10 (0%)	12 (0%)	15 (0%)	11 (0%)	7 (0%)	11 (0%)	17 (0%)	17 (0%)	15 (0%)	22 (0%)	17 (0%)
6	1 (0%)	0	0	0	0	0	0	0	0	0	1 (0%)	0	0	0
ED Glasgow Coma Scale														
13	31,585 (4%)	1278 (5%)	1713 (5%)	1847 (4%)	1983 (4%)	2205 (4%)	2343 (4%)	2376 (4%)	2512 (4%)	2769 (4%)	2975 (4%)	3032 (4%)	3208 (4%)	3344 (4%)
14	138,074 (18%)	4762 (19%)	6508 (18%)	7718 (18%)	8297 (18%)	9289 (18%)	9779 (17%)	10,268 (18%)	11,084 (18%)	12,000 (18%)	13,031 (18%)	14,246 (19%)	15,101 (19%)	15,991 (19%)
15	588,607 (78%)	19,656 (76%)	28,068 (77%)	33,770 (78%)	36,961 (78%)	40,816 (78%)	43,872 (78%)	45,570 (78%)	48,375 (78%)	52,425 (78%)	54,465 (77%)	58,349 (77%)	61,869 (77%)	64,411 (77%)
Injury type														
Blunt	735,486 (97%)	24,540 (96%)	34,814 (96%)	41,750 (96%)	45,435 (96%)	50,315 (96%)	54,128 (97%)	56,159 (96%)	59,605 (96%)	64,998 (97%)	68,772 (98%)	74,065 (98%)	78,682 (98%)	82,223 (98%)
Burn	141 (0%)	5 (0%)	11 (0%)	14 (0%)	10 (0%)	18 (0%)	10 (0%)	8 (0%)	9 (0%)	13 (0%)	10 (0%)	12 (0%)	10 (0%)	11 (0%)
Other/unspecified	18,319 (2%)	926 (4%)	1147 (3%)	1289 (3%)	1485 (3%)	1690 (3%)	1516 (3%)	1743 (3%)	2083 (3%)	1821 (3%)	1304 (2%)	1142 (2%)	1080 (1%)	1093 (1%)
Penetrating	4320 (1%)	225 (1%)	317 (1%)	282 (1%)	311 (1%)	287 (1%)	340 (1%)	304 (1%)	274 (0.4%)	362 (1%)	385 (1%)	408 (1%)	406 (1%)	419 (1%)
Intent of injury														
Assault	50,104 (7%)	2453 (10%)	3330 (9%)	3642 (8%)	3749 (8%)	3916 (7%)	4151 (7%)	3826 (7%)	3838 (6%)	4010 (6%)	4404 (6%)	4307 (6%)	4199 (5%)	4279 (5%)
Other	592 (0.1%)	24 (0.1%)	48 (0.1%)	40 (0.1%)	41 (0.1%)	59 (0.1%)	61 (0.1%)	79 (0.1%)	52 (0.1%)	60 (0.1%)	30 (0%)	26 (0%)	40 (0%)	32 (0%)
Self-inflicted	2036 (0.3%)	67 (0.3%)	96 (0.3%)	133 (0.3%)	134 (0.3%)	140 (0.3%)	157 (0.3%)	169 (0.3%)	164 (0.3%)	198 (0.3%)	205 (0.3%)	197 (0.3%)	183 (0.2%)	193 (0.2%)
Undetermined	5486 (1%)	173 (1%)	142 (0.4%)	168 (0.4%)	271 (1%)	442 (1%)	183 (0.3%)	465 (1%)	870 (1%)	664 (1%)	584 (1%)	530 (1%)	481 (1%)	513 (1%)
Unintentional	700,048 (92%)	22,979 (89%)	32,673 (90%)	39,352 (91%)	43,046 (91%)	47,753 (91%)	51,442 (92%)	53,675 (92%)	57,047 (92%)	62,262 (93%)	65,248 (93%)	70,567 (93%)	75,275 (94%)	78,729 (94%)
Coagulopathy	111,683 (15%)	1260 (5%)	2368 (7%)	3297 (8%)	4155 (9%)	5488 (10%)	6288 (11%)	7616 (13%)	8667 (14%)	10,547 (16%)	11,562 (16%)	14,092 (19%)	17,082 (21%)	19,261 (23%)
Systolic BP, mm Hg, mean (SD)	144.65 (26.95)	143.63 (27.24)	143.6 (27.29)	143.71 (27.17)	144.01 (27.15)	143.88 (27)	144.05 (26.83)	144.62 (26.67)	144.88 (26.89)	145.02 (26.83)	144.65 (26.67)	145.29 (26.95)	145.44 (26.95)	145.35 (27.02)
Pulse, beats/min, mean (SD)	85.02 (18.06)	87.17 (19.08)	86.67 (18.57)	86.04 (18.53)	85.48 (18.5)	85.38 (18.27)	85.41 (18.17)	84.99 (18.05)	84.53 (17.87)	84.82 (17.84)	84.8 (17.88)	84.55 (17.83)	84.35 (17.75)	84.16 (17.71)
Respiratory rate, breaths/min, mean (SD)	18.36 (4.01)	18.9 (4.21)	18.72 (4.16)	18.6 (4.13)	18.52 (4.1)	18.48 (4.17)	18.41 (4.18)	18.35 (4.14)	18.28 (4.13)	18.24 (3.86)	18.24 (3.91)	18.19 (3.79)	18.23 (3.84)	18.25 (3.87)
Injury Severity Scale, mean (SD)	14.65 (7.74)	18.04 (7.84)	17.05 (7.87)	16.65 (7.81)	15.55 (7.64)	14.93 (7.5)	14.74 (7.64)	14.39 (7.55)	14.02 (7.63)	13.87 (7.56)	13.95 (7.68)	14.04 (7.67)	13.95 (7.69)	14.01 (7.69)

SD, standard deviation; ACS, American College of Surgeons; NA, not applicable; MVT, motor vehicle transportation; ED, emergency department; BP, blood pressure.

Distribution of race, hospital bed size, number of hemorrhages, GCS, injury type, and vital signs did not notably change over the 13-year study period. Additionally, Supplementary Figure S2 shows a shifting of patient ages across the 13-year study period, such that there were fewer younger patients and more older patients in 2019, compared to 2007; this was true for all ICH types. Supplementary Table S1 shows demographic, injury, and hospital data stratified by ICH type.

There were 49,220 (7.38%) patients with a documented neurosurgical intervention. Each ICH type had a different proportion of patients receiving at least one neurosurgical intervention: isolated SDHs (13.85%), isolated EDHs (11.06%), multiple hemorrhage types (7.52%), isolated unspecified hemorrhages (2.14%), isolated lacerations/contusions (1.86%), and isolated SAHs (0.80%). Table 2 describes differences in demographic, hospital, and injury characteristics between patients who did and did not receive a neurosurgical intervention. Those receiving a neurosurgical intervention were older, more often male, paying with Medicare, transferred from an outside hospital, sustained a fall-related injury, had pre-injury coagulopathy, and worse emergency department GCS, systolic blood pressure, pulse, and injury severity score values; there were no notable differences between neurosurgical intervention groups in race, hospital bed size, trauma designation level, number of ICHs, injury type, intent of injury, or respiratory rate. Table 3 compares multiple outcome characteristics between ICH types and suggests differences in the timing and number of neurosurgical interventions and hospital length of stay. Patients with multiple ICH types had the longest hospital length of stay, largest number of neurosurgical procedures, and the second longest mean duration from hospital admission to first neurosurgical intervention.

**Table 2.** Demographic, Hospital, and Injury Characteristics of Study Population by Neurosurgical Intervention

Variable,* n *(%)	Overall	No neurosurgery	Neurosurgery
	666,842 (100%)	617,622 (93%)	49,220 (7%)
Year^a^			
2007	16,433 (2%)	15,015 (91%)	1418 (9%)
2008	27,381 (4%)	25,159 (92%)	2222 (8%)
2009	34,201 (5%)	31,515 (92%)	2686 (8%)
2010	38,626 (6%)	35,544 (92%)	3082 (8%)
2011	45,034 (7%)	41,515 (92%)	3519 (8%)
2012	48,460 (7%)	44,890 (93%)	3570 (7%)
2013	50,562 (8%)	46,877 (93%)	3685 (7%)
2014	54,330 (8%)	50,323 (93%)	4007 (7%)
2015	60,112 (9%)	55,798 (93%)	4314 (7%)
2016	65,646 (10%)	61,029 (93%)	4617 (7%)
2017	71,139 (11%)	66,108 (93%)	5031 (7%)
2018	75,904 (11%)	70,521 (93%)	5383 (7%)
2019	79,014 (12%)	73,328 (93%)	5686 (7%)
Age, years, mean (SD)	63.30 (19.44)	62.93 (19.63)	67.91 (16.26)
Male	383,749 (58%)	351,063 (57%)	32,686 (66%)
Race			
Asian	16,575 (2%)	15,248 (2%)	1327 (3%)
Black	60,233 (9%)	55,072 (9%)	5161 (10%)
Other	50,897 (8%)	47,012 (8%)	3885 (8%)
White	539,137 (81%)	500,290 (81%)	38,847 (79%)
Payment method			
Medicaid	55,388 (8%)	51,141 (8%)	4247 (9%)
Medicare	323,920 (49%)	295,173 (48%)	28,747 (58%)
Other	58,654 (9%)	55,320 (9%)	3334 (7%)
Private/commercial	170,868 (26%)	160,904 (26%)	9964 (20%)
Self	58,012 (9%)	55,084 (9%)	2928 (6%)
Interhospital transfer	257,314 (39%)	234,557 (38%)	22,757 (46%)
Hospital beds			
≤200	42,676 (6%)	40,546 (7%)	2130 (4%)
201–400	185,132 (28%)	171,800 (28%)	13,332 (27%)
401–600	186,245 (28%)	171,393 (28%)	14,852 (30%)
>600	252,789 (38%)	233,883 (38%)	18,906 (38%)
ACS trauma-level designation			
1	251,129 (38%)	231,762 (38%)	19,367 (39%)
2	154,662 (23%)	142,821 (23%)	11,841 (24%)
3 or 4	23,492 (4%)	22,584 (4%)	908 (2%)
NA/undesignated	237,559 (36%)	220,455 (36%)	17,104 (35%)
Mechanism of injury			
Fall	478,313 (63%)	435,080 (62%)	43,233 (77%)
MVT motorcyclist	25,769 (3%)	25,052 (4%)	717 (1%)
MVT occupant	110,891 (15%)	107,057 (15%)	3834 (7%)
Other	72,633 (10%)	68,354 (10%)	4279 (8%)
Struck by, again	51,071 (7%)	47,829 (7%)	3242 (6%)
Transport, other	19,589 (3%)	18,891 (3%)	698 (1%)
No. of intracranial hemorrhages			
1	577,050 (76%)	534,782 (76%)	42,268 (75%)
2	143,710 (19%)	134,076 (19%)	9634 (17%)
3	33,401 (4%)	30,037 (4%)	3364 (6%)
4	3924 (1%)	3243 (0.5%)	681 (1%)
5	180 (0%)	124 (0%)	56 (0.1%)
6	1 (0%)	1 (0%)	0
ED Glasgow Coma Scale			
13	31,585 (4%)	27,675 (4%)	3910 (7%)
14	138,074 (18%)	125,417 (18%)	12,657 (23%)
15	588,607 (78%)	549,171 (78%)	39,436 (70%)
Injury type			
Blunt	735,486 (97%)	681,733 (97%)	53,753 (96%)
Burn	141 (0%)	137 (0%)	4 (0%)
Other/unspecified	18,319 (2%)	16,500 (2%)	1819 (3%)
Penetrating	4320 (1%)	3893 (1%)	427 (1%)
Intent of injury			
Assault	50,104 (7%)	47,533 (7%)	2571 (5%)
Other	592 (0.1%)	561 (0.1%)	31 (0.1%)
Self-inflicted	2036 (0.3%)	1862 (0.3%)	174 (0.3%)
Undetermined	5486 (1%)	4891 (1%)	595 (1%)
Unintentional	700,048 (92%)	647,416 (92%)	52,632 (94%)
Coagulopathy	111,683 (15%)	100,331 (14%)	11,352 (20%)
Systolic BP, mm Hg, mean (SD)	144.65 (26.95)	144.32 (26.92)	148.73 (26.97)
Pulse, beats/min, mean (SD)	85.02 (18.06)	85.27 (18.04)	81.83 (18.09)
Respiratory rate, breaths/min, mean (SD)	18.36 (4.01)	18.37 (4.01)	18.18 (3.92)
Injury Severity Scale, mean (SD)	14.65 (7.74)	14.15 (7.54)	20.97 (7.49)

^a^ Row percentages add to 100%.

SD, standard deviation; ACS, American College of Surgeons; NA, not applicable; MVT, motor vehicle transportation; ED, emergency department; BP, blood pressure.

**Table 3.** Outcome Characteristics of Study Population by Intracranial Hemorrhage Type (*n* = 666,842)

Variable,* n *(%)	Overall	Isolated EDH	Isolated lacerations/contusion	Multiple hemorrhages	Isolated SAH	Isolated SDH	Isolated unspecified
	666,842 (100%)	7779 (1%)	43,695 (7%)	159,220 (24%)	157,414 (24%)	238,419 (36%)	60,315 (9%)
Hospital length of stay, days, mean (SD)	5.82 (7.47)	6.87 (9.1)	5.05 (6.98)	7.21 (8.78)	4.98 (6.89)	5.66 (6.78)	5.44 (7.33)
Neurosurgical procedure	49,220 (7%)	860 (11%)	811 (2%)	11,973 (8%)	1264 (1%)	33,020 (14%)	1292 (2%)
No. of neurosurgical procedures							
1	30,504 (62%)	648 (75%)	482 (59%)	6145 (51%)	916 (72%)	21,497 (65%)	816 (63%)
2	12,183 (25%)	156 (18%)	197 (24%)	3353 (28%)	242 (19%)	7913 (24%)	322 (25%)
≥3	6533 (13%)	56 (7%)	132 (16%)	2475 (21%)	106 (8%)	3610 (11%)	154 (12%)
Days to first neurosurgical procedure, mean (SD)	2.17 (3.7)	1.32 (1.33)	1.87 (3.1)	2.25 (5.42)	2.58 (3.95)	1.95 (2.68)	2.19 (3.93)
Death/hospice	28,100 (4%)	233 (3.00%)	810 (1.85%)	11,768 (7.39%)	3258 (2.07%)	9856 (4.13%)	2175 (3.61%)

EDH, epidural hemorrhage; SAH, subarachnoid hemorrhage; SDH, subdural hemorrhage; SD, standard deviation.

**FIG. 2.**
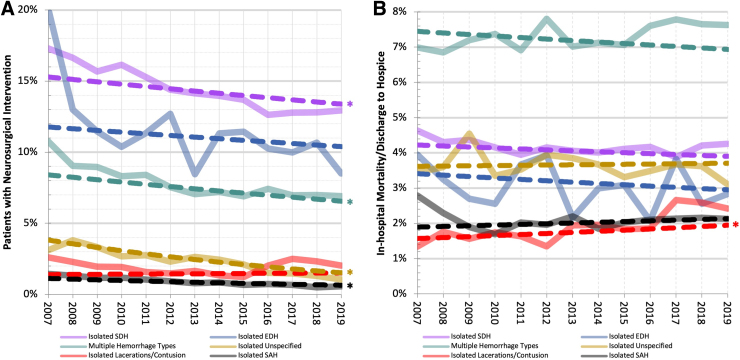
Observed and fully adjusted long-term outcome trends by year and ICH type. (**A**) Long-term trends in percentage of complicated mTBI patients who received a neurosurgical intervention. Solid lines indicate observed data, and dashed lines indicate trends adjusted for all 14 demographic, injury, and hospital characteristics. There were significant long-term declines in the adjusted percentage of patients with isolated subdural, subarachnoid, unspecified, and multiple hemorrhage types who received a neurosurgical procedure. (**B**) Long-term trends in the percentage of patients with an in-hospital mortality or discharge to hospice. Most ICH types did not have a significant long-term change in the adjusted percentage of patients suffering from an in-hospital mortality or discharge to hospice; the only exception was the significant positive trend observed in patients with isolated lacerations/contusions. Asterisks indicate statistically significant adjusted slopes (*p* < 0.05). Adjusted slope lines from the random-effect model were plotted with an intercept centered at 2013 that aligned to the average 5-year observed outcome percentage during 2011–2015. EDH, epidural hemorrhage; ICH, intracranial hemorrhage; SAH, subarachnoid hemorrhage; SDH, subdural hemorrhage.

According to the mixed-effects multi-variable logistic regression model, after adjustment for demographic, injury, and hospital characteristics, all ICH types had significant decreases in odds of neurosurgical intervention over time, except isolated EDHs and isolated lacerations and contusions (Table 4; Fig. 2A). Results from model 5 suggest that adjustment for the potentially confounding demographic, injury, and hospital variables nominally attenuated the time trends toward the null, compared to unadjusted models (model 2). The largest relative changes in odds of neurosurgical intervention over time occurred for isolated unspecified hemorrhages (−50%) and isolated SAHs (−48%), whereas other hemorrhages had smaller changes (−15% to +2%).

**Table 4.** Neurosurgical Intervention Trends per Decade by Intracranial Hemorrhage Type, Adjusting for Demographics, Hospital Characteristics, and Injury Characteristics

Odds ratios of neurosurgical intervention per 10 years [95% CI]	N	Model 1 (time)	Model 2 (time)	Model 3 (M2 + demographics)	Model 4 (M3 + hospital characteristics)	Model 5 (M4 + injury characteristics)
Year (continuous)	666,842	1.03 [0.95, 1.11]				
Stratified by ICH type						
Isolated SDH	238,419		0.87 [0.80, 0.96]	0.81 [0.75, 0.89]	0.77 [0.71, 0.84]	0.85 [0.78, 0.93]
Multiple hemorrhages	159,220		0.82 [0.75, 0.89]	0.77 [0.71, 0.84]	0.77 [0.71, 0.83]	0.79 [0.73, 0.86]
Isolated SAH	157,414		0.50 [0.40, 0.63]	0.50 [0.40, 0.63]	0.49 [0.40, 0.61]	0.63 [0.51, 0.77]
Isolated unspecified	60,315		0.40 [0.32, 0.50]	0.38 [0.30, 0.47]	0.38 [0.31, 0.46]	0.50 [0.41, 0.62]
Isolated Lac/contusion	43,695		0.97 [0.70, 1.34]	0.87 [0.63, 1.21]	0.90 [0.65, 1.24]	1.03 [0.75, 1.41]
Isolated EDH	7779		0.73 [0.56, 0.93]	0.79 [0.62, 1.02]	0.80 [0.62, 1.02]	0.87 [0.68, 1.12]

Demographic variables: age, sex, race, and payment method. Hospital characteristics: bed size and American College of Surgeons trauma designation level. Injury characteristics: interhospital transfer, mechanism of injury, emergency department Glasgow Coma Scale, Injury Severity Score, injury type (e.g., blunt), intent of injury, coagulopathy, systolic emergency department blood pressure, pulse, and respiratory rate.

OR, odds ratio; CI, confidence interval; ICH, intracranial hemorrhage; EDH, epidural hemorrhage; SAH, subarachnoid hemorrhage; SDH, subdural hemorrhage.

Overall, 4.2% (*n* = 28,100) of the study population suffered an in-hospital death or was discharged to hospice. Table 3 shows that the largest death/hospice proportion was observed in patients presenting with multiple hemorrhage types (7.4%), and the lowest was observed in patients presenting with isolated contusions (1.9%). The mixed-effects logistic regression model suggested that after adjustment for the 15 demographic, injury, and hospital characteristics, the odds ratio [95% confidence interval] for in-hospital death/hospice (comparing years 2019–2007) was 0.80 [0.47, 1.35] for isolated EDHs, 1.41 [1.05, 1.91] for isolated lacerations and contusions, 0.91 [0.82, 1.00] for multiple hemorrhage types, 1.17 [0.99, 1.37] for isolated SAHs, 0.89 [0.80, 1.00] for isolated SDHs, and 1.04 [0.86, 1.25] for isolated unspecified hemorrhages.

## Discussion

This study used 13 years of patient- and hospital-level data from the NTDB to characterize recent long-term trends in neurosurgical intervention and mortality in a diverse patient population with complicated mTBI in the United States. These results suggest significant long-term declines in the proportion of patients with complicated mTBI receiving a neurosurgical intervention. Simultaneously, odds of in-hospital mortality or discharge to hospice did not significantly change over time for all ICH types, except isolated lacerations and contusions, which showed a significant increase over time.

These study data suggest that both the patient population suffering from complicated mTBI and the neurosurgical treatment of this injury has clearly changed over the previous decade. According to all the demographic data, this complicated mTBI population is one increasingly comprised of older adults, an observation similarly noted in a 20-year Belgian TBI study.^17^ An increase in the older adult population is in agreement with the observed increases in absolute number of patients per hospital presenting with isolated subdural, subarachnoid, and multiple hemorrhage types (Fig. 1B); these three ICH types had the highest average age (Supplementary Table S1). Separately, we observed stable longitudinal trends in average number of neurosurgical interventions per hospital for all ICH types except isolated SDH, which showed an increasing trend over time (Fig. 1B). If overall neurosurgical practice patterns remained the same, we would have expected to see no change in relative odds of neurosurgical intervention over time. We found the opposite.

There were significant decreases in the proportion of patients in nearly every ICH category who received a neurosurgical intervention, after adjusting for potentially confounding variables. Therefore, despite the increase in absolute number of patients per hospital presenting with mTBI and isolated subdural, subarachnoid, unspecified, and multiple types of hemorrhages, neurosurgeons were surgically intervening relatively less frequently. The reasons for this relationship are unknown.

It could be suggested that the changing demographics, injury characteristics, and varying hospital participation over time are contributing to the increasing number of patients and decreasing proportion receiving neurosurgical intervention. However, according to the multi-variable mixed-effects logistic regression model, the declining relative neurosurgical intervention trends are minimally explained by the various demographic, injury, and hospital characteristics adjusted for in the models. Other reasons explaining this observation might include changing hemorrhage characteristics over time (e.g., more frequent identification of smaller, less serious hemorrhages), changing practice patterns over time (e.g., using watch-and-wait approaches more frequently), or changing patient/family decision making over time (e.g., increasing frequency of refusing neurosurgical interventions).

We posit that a combination of changing practice patterns and hemorrhage characteristics over time could explain the decreasing use of neurosurgical interventions to manage patients with complicated mTBI. There have been numerous publications in recent years citing the infrequent need for neurosurgical intervention in the vast majority of patients with complicated mTBI.^9,18–23^ We also understand that improved resolution of head imaging over time has allowed for the detection of smaller hemorrhages^24,25^ and that smaller ICHs predict lower risk of neurosurgical intervention.^26–30^ These facts suggest that previously low neurosurgical intervention rates might be driven even lower by the discovery of smaller, non-serious hemorrhages that carry a lower risk of neurosurgical intervention. Regarding the neurosurgical workforce, multiple national societies have sounded the alarm about the lack of the number of neurosurgeons and their increasing reluctance to take a trauma call.^31,32^ Thus, the decreasing proportion of patients with complicated mTBI receiving neurosurgical intervention could indicate that neurosurgeons burdened by high patient volumes are becoming more selective about which patients are surgically managed.

Importantly, we observed that the relative decrease in neurosurgical interventions in patients with complicated mTBI was simultaneous with no change in probability of in-hospital mortality or discharge to hospice for all ICH types except isolated lacerations and contusions. This observation supported the possibility that an increase in less serious hemorrhages is occurring, rather than an increase in refusal of neurosurgical interventions. It also supported the hypothesis that neurosurgeons are becoming more selective in which ICHs are surgically managed. Worth noting is that isolated lacerations and contusions were the only hemorrhage type to have a nearly unchanging 10-year trend in the adjusted odds of neurosurgical intervention (odds ratio = 1.02) and were the only hemorrhage type to have a borderline significant increase in odds of in-hospital mortality or discharge to hospice (+40%). This large relative increase in adjusted odds is partially a result of this hemorrhage type having a low absolute value of death/hospice in 2007 (∼1.01%), with a small absolute increase of 0.37% in 2019, resulting in a large relative increase in odds (Fig. 2B).

It is also important to note that isolated lacerations and contusions were the only hemorrhage type to show a notable decrease over time in average number of patients per hospital. A potential explanation for the above-mentioned association is that less severe lacerations and contusions have become more infrequent, whereas severe lacerations and contusions become more frequent and carry an increased risk of death or hospice. To move from conjecture to evidence-based conclusions, longitudinal studies must track detailed hemorrhage characteristics (e.g., size and location) in conjunction with neurosurgical interventions over time.

This study has several limitations. First, the NTDB is voluntary and a convenience sample of trauma patients across the United States. It is not able to provide national prevalence estimates, nor were we in control of the quality of data submitted by hospitals. The granularity of the data in the NTDB is also limited, and although it contains numerous patient- and hospital-level variables, it does not allow us to rule out the possibility for unmeasured confounding (e.g., severity/size of ICH) and residual confounding to impact our results. Nevertheless, the NTDB is also a strength of the study, given that it is the largest national database for trauma admissions in the United States and provides important longitudinal patient- and hospital-level characteristics. The methods we chose to analyze the long-term trends in neurosurgical intervention in the United States capitalized on the rich data included in the NTDB. These methods weighted the influence of individual hospital trends based on the variability of their data; hospitals with less variability/data were weighted more than hospitals with more variability/data. This methodology decreases the influence of “outlier” hospitals on overall trends over time. Another strength of the study is that each ICH type was examined separately, avoiding the overgeneralization of examining all ICH types as one. As such, we were able to highlight the uniqueness of each ICH type with respect to their demographics, injury characteristics, trends over time, and outcome measures.

## Conclusion

This study presents important long-term national trends of neurosurgical interventions in trauma patients with complicated mTBI. We observed significant declines in use of neurosurgical interventions to manage nearly all ICH types. Additionally, odds of in-hospital mortality/hospice remained stable across the 13-year study period. In the upcoming decades, the number of older adults at risk of suffering a complicated mTBI will nearly double, increasing patient burden on hospitals and neurosurgical departments.^10,33^ This will require more efficient methods of management and potentially a larger neurosurgical workforce. Current trends suggest that the use of neurosurgical interventions has become more efficient in patients with mTBI and ICH, but more research is needed to identify the driving factors and optimize them for the future.

## Supplementary Material

Supplemental data

Supplemental data

Supplemental data

## Abbreviations Used

ACS: American College of Surgeons
EDH: epidural hemorrhage
GCS: Glasgow Coma Scale
ICD-9: International Classification of Diseases, Ninth Revision
ICD-10: International Classification of Diseases, Tenth Revision
ICH: intracranial hemorrhage
ICP: intracranial pressure
ISS: Injury Severity Score
mTBI: mild traumatic brain injury
NTDB: National Trauma Data Bank
SAH: subarachnoid hemorrhage
SDH: subdural hemorrhage
