# Supplementary material for: Significant National Declines in Neurosurgical Intervention for Mild Traumatic Brain Injury with Intracranial Hemorrhage: A 13-Year Review of the National Trauma Data Bank
Source: Neurotrauma Rep. 2023 Mar 17;4(1):137–48. doi: 10.1089/neur.2022.0077 (PMC10024583; doi:10.1089/neur.2022.0077)
Supplement: Supplemental data [file Supp_FigS1.docx]

Supplemental Figure 1: Number of hospitals contributing study data to the NTDB per year.
