# Supplementary material for: Significant National Declines in Neurosurgical Intervention for Mild Traumatic Brain Injury with Intracranial Hemorrhage: A 13-Year Review of the National Trauma Data Bank
Source: Neurotrauma Rep. 2023 Mar 17;4(1):137–48. doi: 10.1089/neur.2022.0077 (PMC10024583; doi:10.1089/neur.2022.0077)
Supplement: Supplementary file 3 [file Supp_TableS1.docx]

TABLE S1. Demographics of study population by intracranial hemorrhage type (n=666,842)

Variable, n(%)	**Overall** 666,842 (100%)	**Isolated EDH** 7,779 (1%)	**Isolated Lacerations/Contusion** 43,695 (7%)	**Multiple Hemorrhages** 159,220 (24%)	**Isolated SAH** 157,414 (24%)	**Isolated SDH** 238,419 (36%)	**Isolated Unspecified** 60,315 (9%)
Year, (obs in full dataset)[a]							
2007 (506,257)	16433 (2%)	304 (2%)	2378 (14%)	4005 (24%)	3625 (22%)	4382 (27%)	1739 (11%)
2008 (633,952)	27381 (4%)	431 (2%)	3517 (13%)	6410 (23%)	6269 (23%)	7917 (29%)	2837 (10%)
2009 (684,460)	34201 (5%)	593 (2%)	3971 (12%)	8144 (24%)	7816 (23%)	10246 (30%)	3431 (10%)
2010 (721,536)	38626 (6%)	626 (2%)	4065 (11%)	8996 (23%)	8857 (23%)	12295 (32%)	3787 (10%)
2011 (783,476)	45034 (7%)	600 (1%)	4341 (10%)	10498 (23%)	10343 (23%)	14858 (33%)	4394 (10%)
2012 (830,774)	48460 (7%)	559 (1%)	3992 (8%)	11208 (23%)	11433 (24%)	16532 (34%)	4736 (10%)
2013 (827,289)	50562 (8%)	556 (1%)	3757 (7%)	11670 (23%)	11777 (23%)	17901 (35%)	4901 (10%)
2014 (858,362)	54330 (8%)	601 (1%)	3362 (6%)	12968 (24%)	12679 (23%)	19540 (36%)	5180 (10%)
2015 (912, 816)	60112 (9%)	647 (1%)	3708 (6%)	14128 (24%)	14171 (24%)	22029 (37%)	5429 (9%)
2016 (963,134)	65646 (10%)	722 (1%)	2903 (4%)	16021 (24%)	15946 (24%)	24494 (37%)	5560 (8%)
2017 (997,970)	71139 (11%)	731 (1%)	2555 (4%)	17536 (25%)	17178 (24%)	27178 (38%)	5961 (8%)
2018 (1,043,736)	75904 (11%)	703 (1%)	2629 (3%)	18312 (24%)	18407 (24%)	29638 (39%)	6215 (8%)
2019 (1,097,190)	79014 (12%)	706 (1%)	2517 (3%)	19324 (24%)	18913 (24%)	31409 (40%)	6145 (8%)
Age, years, mean (SD)	63.3 (19.44)	54.39 (22.35)	52.73 (21.61)	63.72 (19.02)	60.79 (19.35)	67.62 (17.5)	60.49 (21.1)
Male	383749 (58%)	5061 (65%)	28946 (66%)	97274 (61%)	81713 (52%)	133497 (56%)	37258 (62%)
Race							
Asian	16575 (2%)	175 (2%)	879 (2%)	4530 (3%)	4022 (3%)	5929 (2%)	1040 (2%)
Black	60233 (9%)	825 (11%)	5384 (12%)	13616 (9%)	14137 (9%)	20275 (9%)	5996 (10%)
Other	50897 (8%)	745 (10%)	4326 (10%)	12316 (8%)	12985 (8%)	16571 (7%)	3954 (7%)
White	539137 (81%)	6034 (78%)	33106 (76%)	128758 (81%)	126270 (80%)	195644 (82%)	49325 (82%)
Payment method							
Medicaid	55388 (8%)	859 (11%)	4154 (10%)	13802 (9%)	13282 (8%)	18143 (8%)	5148 (9%)
Medicare	323920 (49%)	2614 (34%)	12900 (30%)	77278 (49%)	66570 (42%)	137894 (58%)	26664 (44%)
Other	58654 (9%)	971 (12%)	6716 (15%)	13418 (8%)	15125 (10%)	16404 (7%)	6020 (10%)
Private/commercial	170868 (26%)	2320 (30%)	13228 (30%)	41211 (26%)	47002 (30%)	50821 (21%)	16286 (27%)
Self	58012 (9%)	1015 (13%)	6697 (15%)	13511 (8%)	15435 (10%)	15157 (6%)	6197 (10%)
Inter-hospital transfer	257314 (39%)	2805 (36%)	12783 (29%)	60914 (38%)	56686 (36%)	103032 (43%)	21094 (35%)
Hospital bedsize							
≤200	42676 (6%)	547 (7%)	2949 (7%)	8297 (5%)	10443 (7%)	15669 (7%)	4771 (8%)
201 to 400	185132 (28%)	2069 (27%)	13410 (31%)	40995 (26%)	43389 (28%)	68347 (29%)	16922 (28%)
401 to 600	186245 (28%)	2210 (28%)	12325 (28%)	46417 (29%)	43140 (27%)	65725 (28%)	16428 (27%)
>600	252789 (38%)	2953 (38%)	15011 (34%)	63511 (40%)	60442 (38%)	88678 (37%)	22194 (37%)
ACS Trauma level designation							
1	251129 (38%)	2931 (38%)	13375 (31%)	64485 (41%)	61940 (39%)	88036 (37%)	20362 (34%)
2	154662 (23%)	1566 (20%)	12389 (28%)	35756 (22%)	35473 (23%)	56857 (24%)	12621 (21%)
3 or 4	23492 (4%)	295 (4%)	1335 (3%)	3995 (3%)	5647 (4%)	9376 (4%)	2844 (5%)
NA/undesignated	237559 (36%)	2987 (38%)	16596 (38%)	54984 (35%)	54354 (35%)	84150 (35%)	24488 (41%)
Mechanism of injury							
Fall	478313 (63%)	4676 (52%)	23252 (45%)	114931 (63%)	101270 (56%)	194270 (73%)	39914 (58%)
MVT Motorcyclist	25769 (3%)	332 (4%)	3032 (6%)	6870 (4%)	8116 (5%)	4511 (2%)	2908 (4%)
MVT Occupant	110891 (15%)	1734 (19%)	11467 (22%)	23786 (13%)	32646 (18%)	28860 (11%)	12398 (18%)
Other	72633 (10%)	1063 (12%)	7241 (14%)	19018 (10%)	20001 (11%)	18577 (7%)	6733 (10%)
Struck by, again	51071 (7%)	890 (10%)	4825 (9%)	12010 (7%)	12175 (7%)	16569 (6%)	4602 (7%)
Transport, other	19589 (3%)	338 (4%)	2415 (5%)	4601 (3%)	5610 (3%)	4320 (2%)	2305 (3%)
Number of intracranial hemorrhages							
1	577050 (76%)	9033 (100%)	52232 (100%)	-	179818 (100%)	267107 (100%)	68860 (100%)
2	143710 (19%)	-	-	143710 (79%)	-	-	-
3	33401 (4%)	-	-	33401 (18%)	-	-	-
4	3924 (1%)	-	-	3924 (2%)	-	-	-
5	180 (0%)	-	-	180 (0.1%)	-	-	-
6	1 (0%)	-	-	1 (0%)	-	-	-
ED Glasgow coma scale							
13	31585 (4%)	291 (3%)	2094 (4%)	11697 (6%)	5925 (3%)	8644 (3%)	2934 (4%)
14	138074 (18%)	1156 (13%)	8867 (17%)	43003 (24%)	29347 (16%)	42823 (16%)	12878 (19%)
15	588607 (78%)	7586 (84%)	41271 (79%)	126516 (70%)	144546 (80%)	215640 (81%)	53048 (77%)
Injury type							
Blunt	735486 (97%)	8646 (96%)	49758 (95%)	175646 (97%)	174677 (97%)	259910 (97%)	66849 (97%)
Burn	141 (0%)	3 (0%)	13 (0%)	26 (0%)	36 (0%)	52 (0%)	11 (0%)
Other/unspecified	18319 (2%)	276 (3%)	1835 (4%)	4367 (2%)	3764 (2%)	6430 (2%)	1647 (2%)
Penetrating	4320 (1%)	108 (1%)	626 (1%)	1177 (1%)	1341 (1%)	715 (0.3%)	353 (1%)
Intent of injury							
Assault	50104 (7%)	868 (10%)	5282 (10%)	12408 (7%)	12446 (7%)	14843 (6%)	4257 (6%)
Other	592 (0.1%)	18 (0.2%)	74 (0.1%)	111 (0.1%)	143 (0.1%)	183 (0.1%)	63 (0.1%)
Self-inflicted	2036 (0.3%)	54 (1%)	221 (0.4%)	560 (0.3%)	501 (0.3%)	494 (0.2%)	206 (0.3%)
Undetermined	5486 (1%)	60 (1%)	469 (1%)	1258 (1%)	1101 (1%)	2105 (1%)	493 (1%)
Unintentional	700048 (92%)	8033 (89%)	46186 (88%)	166879 (92%)	165627 (92%)	249482 (93%)	63841 (93%)
Coagulopathy	111683 (15%)	802 (9%)	3611 (7%)	29661 (16%)	22584 (13%)	45899 (17%)	9126 (13%)
Systolic BP, mmHg, mean (SD)	144.65 (26.95)	141.24 (26.49)	141.51 (25.72)	145.18 (27.21)	141.7 (26.18)	147.03 (27.22)	144.52 (27.16)
Pulse, beats/min, mean (SD)	85.02 (18.06)	84.87 (18.27)	87.16 (18.64)	85.67 (18.34)	85.96 (18.17)	83.37 (17.48)	85.63 (18.32)
Respiratory rate, breaths/min, mea	18.36 (4.01)	18.54 (4.07)	18.58 (4.28)	18.49 (4.16)	18.41 (4.1)	18.16 (3.76)	18.45 (4.05)
Injury severity scale, mean (SD)	14.65 (7.74)	18.32 (8.34)	13.29 (7)	17.2 (7.78)	9.32 (6.38)	16.67 (6.71)	14.54 (7.93)

EDH, epidural hemorrhage; SAH, subarachmnoid hemorrhage; SDH, subdural hemorrhage; d, days; SD, standard deviation.

[a] Row percentages add to 100%.
